# Provocation CT-Based Analysis for Diagnosis of Hip and Knee Arthroplasty Aseptic Loosening: Where Are We at? A Systematic Review of Clinical Trials

**DOI:** 10.3390/jcm14144865

**Published:** 2025-07-09

**Authors:** Lorenzo Impieri, Riccardo Uras, Marco Pilone, Andrea Pezzi, Giacomo Folli, Luigi Impieri, Nicolò Rossi

**Affiliations:** 1 Residency Program in Orthopedics and Traumatology, University of Milan, 20122 Milan, Italy; riccardo.uras@unimi.it (R.U.); marco.pilone@unimi.it (M.P.); andrea.pezzi@unimi.it (A.P.); nicolo.rossi@grupposandonato.it (N.R.); 2 Istituto Clinico San Siro, 20148 Milan, Italy; giacomo.folli@grupposandonato.it; 3 Residency Program in Radiology and Imaging, University of Campania “Luigi Vanvitelli”, 80138 Naples, Italy; luigi.impieri@studenti.unicampania.it; 4 IRCCS Ospedale Galeazzi Sant’Ambrogio, 20157 Milan, Italy

**Keywords:** hip, knee, arthroplasty, aseptic loosening, provocation CT Analysis

## Abstract

**Background/Objectives:** Aseptic loosening is a major challenge in hip and knee arthroplasty. While radiostereometric analysis (RSA) is the gold standard for detecting early migration, it is static, costly, and requires metal beads. Provocation CT-based analysis studies implants under physiological stresses and offers a marker-free alternative with comparable accuracy. This systematic review evaluates its effectiveness, cost, and role in orthopedic imaging. **Methods:** A systematic review was conducted following the Preferred Reporting Items for Systematic Review and Meta-Analyses (PRISMA) guidelines. Three databases were searched, with no date restrictions, using keywords related to the research area. The risk of bias was assessed using the RoB-1 tool. **Results:** The initial search identified 42 studies, with 6 ultimately included in the review. These studies involved 198 patients with an average age of 65.0 years. Provocation CT demonstrated higher sensitivity and specificity than standard radiographs, particularly in cases with inconclusive X-rays. Additionally, the radiation dose for CT scans varied across studies, with effective doses ranging from 0.2 mSv to 4.5 mSv per scan. Compared to X-ray, CT-based methods showed comparable or superior performance in motion detection, though direct clinical comparisons with RSA remain lacking. **Conclusions:** Provocation CT-based analysis is a valuable diagnostic tool for early detection of implant loosening, offering a potentially feasible, accurate, and cost-effective alternative to traditional methods. However, standardized protocols, broader economic evaluations, and prospective multicenter trials are needed to confirm its routine clinical applicability.

## 1. Introduction

Total hip arthroplasty (THA) and total knee arthroplasty (TKA) are among the most successful orthopedic procedures, significantly improving function and quality of life in patients with degenerative joint diseases [1,2]. However, despite advances in implant design and surgical techniques, implant loosening remains a major cause of failure, often leading to revision surgery, which is more invasive and carries higher risks than primary arthroplasty [3,4]. The ability to detect early micromotion of an implant is crucial, as excessive migration within the first two years is a strong predictor of long-term instability and failure [5,6,7]. Traditional imaging techniques, such as standard radiography, usually depend on disclosures of linear osteolysis that are hard to diagnose and often lack the sensitivity to detect small-scale implant migration, making it difficult to identify patients at risk before significant complications develop [8,9].

Historically, radio stereometric analysis (RSA) has been the gold standard for assessing early implant migration [10,11]. RSA provides highly accurate measurements using tantalum markers embedded in the bone and implant, allowing for precise tracking of movement over time [12]. However, its clinical application is limited by the need for marker placement, specialized equipment, and high costs, making it impractical for routine use outside of research settings [13]. As a result, alternative imaging methods have been developed to offer similar precision in a more accessible and less invasive manner [14]. Provocation CT-based analysis has emerged as a promising alternative to RSA, leveraging computed tomography (CT) imaging and advanced software algorithms to assess implant migration without requiring pre-implanted markers [15]. Unlike traditional static imaging, provocation CT-based analysis incorporates multiple sequential CT scans acquired under different loading conditions, such as forced internal rotation and forced external rotation, allowing for a dynamic evaluation of implant movement relative to the surrounding bone [16]. This method does not rely on a single pair of images but rather registers multiple acquisitions to assess the relative displacement of the implant over time [17]. This approach allows provocation CT-based analysis to assess implant stability under conditions that mimic real-life joint loading, improving the detection of early aseptic loosening [18]. This ability to detect micromotion under stress potentially makes this CT analysis particularly useful in cases where standard X-rays fail to reveal early signs of loosening, enabling earlier intervention and reducing the risk of catastrophic implant failure. Since this diagnostic method is not routinely used in clinical practice yet, through this systematic review we aim to critically evaluate the evidence on the application of provocation CT-based analysis in hip and knee arthroplasty. Specifically, we seek to determine its accuracy in detecting implant migration, compare its performance with RSA and standard radiographic methods, and assess its cost-effectiveness and feasibility.

## 2. Materials and Methods

**Figure 1:**
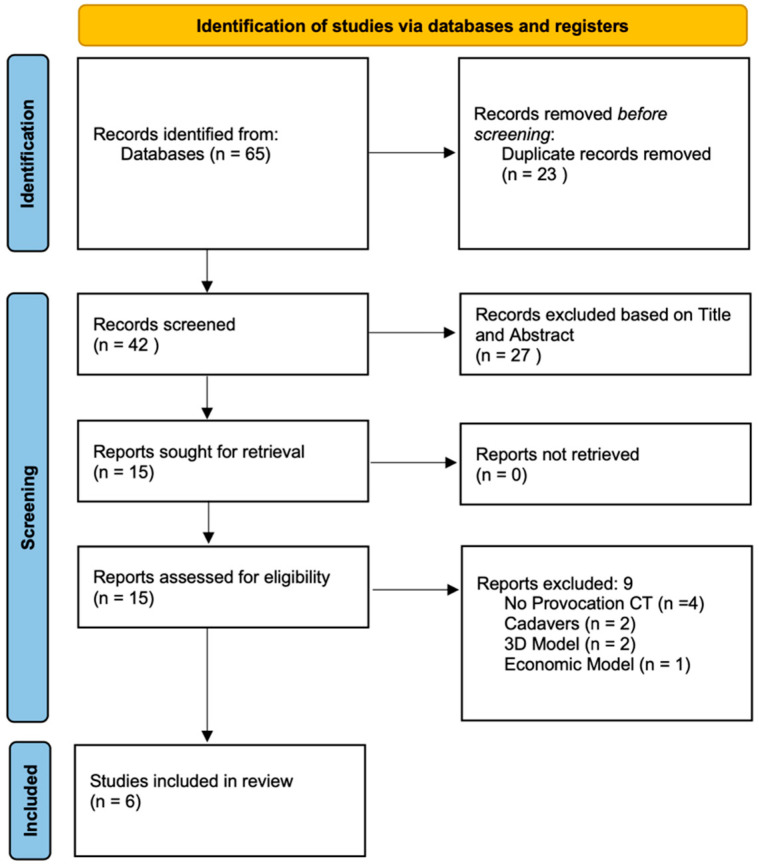
PRISMA flow chart.

This systematic review was based on the Preferred Reporting Items for Systematic Review and Meta-Analyses [19] (Figure 1) checklist structure and followed the recommendations of the Enhancing the Quality and Transparency of Health Research Network. Moreover, this systematic review was registered in the International Prospective Register of Systematic Reviews (PROSPERO: 1049224).

### 2.1. Focus Question

This systematic review was conducted to examine the current evidence on the usage of provocation CT-based analysis for the detection of knee and hip arthroplasty aseptic loosening.

### 2.2. Search Strategy

A literature search was performed using three databases (PubMed, Embase, and Scopus) with no date restriction but limited to publications in the English language. The search was carried out up to 21 December 2024 and was performed with medical subject heading terms/entry terms such as “provocation computer tomography”, “provocation CT”, “implant movement analysis”, “arthroplasty”, “aseptic loosening”, “hip”, and “knee”. In addition, an independent manual search was conducted using terms adapted for each database, including the grey literature and relevant journals in the field. A manual search was also conducted on the reference lists of relevant review studies. Alerts were established for each database to ensure that the search strategy was up to date.

### 2.3. Eligibility Criteria

The PICO framework [20] was used to target our focus question as follows:

(P) Population: Humans (>18 y.o.) affected by aseptic loosening of hip and knee arthroplasty.
(I) Intervention: Usage of provocation CT-based diagnostic methods.
(C) Comparison: Classical diagnostic methods such as X-rays and RSA.
(O) Outcome: Effectiveness of diagnosis of aseptic loosening.

Included in this systematic review were (1) human clinical studies, (2) articles written in English and (3) published in a peer-reviewed journal with (4) the full text available, (5) in which the use of provocation CT-based analysis in hip and knee arthroplasty for aseptic loosening diagnosis was mentioned.

Studies conducted on animal models or designated as purely in vitro, review articles, expert opinions, abstract-only articles, studies for which the full texts were unavailable, or studies in which provocation computer tomography was used in arthroplasty of other joints were excluded.

### 2.4. Study Selection

For this purpose, all the references retrieved from the databases were imported to the Rayyan Intelligent Systematic Review platform (https://www.rayyan.ai/, accessed on 23 December 2024). Initially, cross-checking eliminated all duplicates, and two reviewers (L.I. and R.U.) independently assessed all titles and abstracts for inclusion using the inclusion criteria described above. In case of a disagreement, a third reviewer (G.F.) was consulted and the final decision was settled by consensus. The kappa coefficient value was calculated to determine inter-reader agreement. Finally, a full-screen process was performed for the remaining articles that met the inclusion and exclusion criteria.

### 2.5. Data Extraction

The following information was recorded: author(s), year of publication, age (years), gender, joint involved, experimental groups, periods of analysis (months), types of diagnostic method used, provocation method, radiation dose, economic data, and main findings. In the case of missing data, one attempt to contact the corresponding author was performed.

### 2.6. Risk of Bias Assessment

The risk of bias was evaluated according to the guidelines of the *Cochrane Handbook for Systematic Reviews of Interventions* [21]. Two reviewers (M.P. and L.I.) independently assessed the risk of bias in the included studies. Since all included studies were non-randomized clinical trials, the Risk of Bias in Non-randomized Studies of Interventions (ROBINS-I) tool [22] was used.

ROBINS-I assesses seven key domains of potential bias in non-RCTs. The first two domains evaluate possible confounding factors and the process of participant selection before the intervention. The third domain assesses the risk of bias in the classification of interventions. The last four domains relate to bias introduced after the intervention: deviations from intended interventions, missing outcome data, the measurement of outcomes, and the selection of the reported results. Each domain was rated as having a risk of bias and categorized as low, moderate, severe, or critical. The overall risk of bias for each study was determined based on the highest level of bias identified across domains. Disagreements between reviewers were resolved by discussion with a third reviewer (G.F.). The ROBINS-I figure was elaborated using the Robvis Software ROBINS-1 V2 (Risk-of-bias VISualization, Riskofbias.info, Bristol, UK) [23].

## 3. Results

### 3.1. Study Selection and Patients’ Characteristics

This systematic review analyzed six studies that investigated the efficacy of provocation CT-based techniques for diagnosing aseptic implant loosening in THA and TKA. These studies collectively included 198 patients, with demographic information reported in Table 1. Sex distribution was explicitly mentioned in four studies (132 patients; 52 males and 80 females). The participants’ median age was approximately 66 years (range reported: 66.0 ± 2.4 years). In total, the reviewed studies evaluated 157 hip implants and 41 knee implants. Four studies specifically addressed THA [18,24,25,26], one study evaluated only TKA [17], and one study evaluated both joints [27]. For more information, see Table 1.

### 3.2. Risk of Bias Assessment

**Figure 2:**
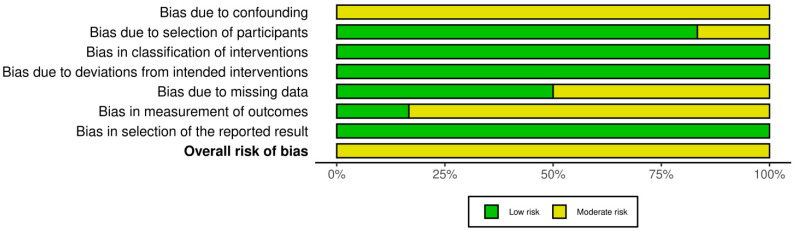
Risk of bias assessment for each domain and overall risk of bias.

**Figure 3:**
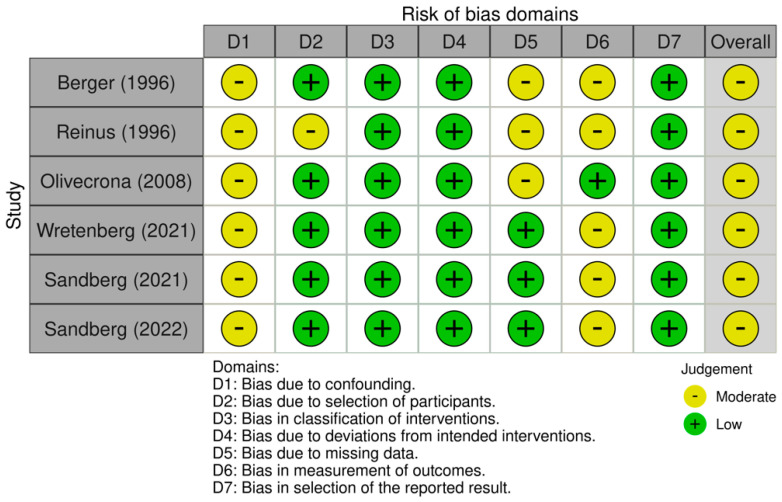
Risk of bias assessment of the included studies [17,18,24,25,26,27].

All the studies included in this systematic review were non-randomized clinical trials and were evaluated using the ROBINS-I risk of bias tool. Of the six studies analyzed, none was rated as having a serious or critical risk of bias in any domain. Most of the studies reported clearly defined eligibility criteria and detailed inclusion protocols, resulting in a low risk of bias for both the selection of participants and the classification of interventions. The domain of confounding consistently showed a moderate risk across all studies, reflecting the non-randomized design and the absence of statistical adjustment for potential baseline differences [28,29,30]. Similarly, a moderate risk of bias was identified in the measurement of outcomes, as the radiological assessments were not conducted in a blinded fashion in most cases, potentially leading to detection bias. In contrast, the domains related to the post-intervention methodology—such as deviations from intended interventions, missing outcome data, and selection of the reported results—were generally judged to carry a low risk, reflecting strong adherence to protocols and complete data reporting in most of the investigations. The overall risk of bias was judged to be moderate in 100% (six out of six) of the included studies. Although these findings underscore the inherent limitations of observational study designs, the overall methodological quality was found to be acceptable and consistent across domains, supporting the credibility of the synthesized results (Figure 2 and Figure 3).

### 3.3. CT Protocols and Radiation Exposure

CT acquisition protocols varied significantly across the included studies, influencing diagnostic performance and radiation exposure (summary in Table 2). Berger et al. [24] in 1996 employed high milliampere settings with maximal internal and external leg rotation and a specialized leg holder; while they optimized metallic artifact reduction, specific radiation doses were not explicitly reported. The CT technique uses a high milliamperage, a 3 s scan time, and 1.5 mm thick sections. Reinus et al. [25], in the same year, used historical standard hip protocols (1990s-era), without specific radiation data, employing torsional stress to evaluate femoral stem loosening. In their study nine patients were examined using a General Electric 9800 scanner (General Electric Medical Systems, Milwaukee, WI, USA) and seven were examined using a General Electric HiSpeed Advantage scanner (General Electric Medical Systems). Images were obtained using a 5 mm collimation and a 5 mm slice thickness.

Olivecrona et al. [18], in 2008, developed a CT Implant Movement Analysis (CT-IMA) protocol consisting of two CT examinations of each hip, using a four-detector-row CT scanner (LightSpeed QX/I; General Electric Medical Systems, Milwaukee, WI, USA). Only the slices around the cup were used for their study. The segment involved 50 slices, acquired with 1.25 mm collimation and a pitch of 3, at 200 mA and 120 kV. Slices were reconstructed at 1.25 mm increments. The x-y pixel size varied from 1.30 to 1.66 mm. The original CTs were reconstructed with a matrix size of 512 × 512, with the number of slices varying between 214 and 309. The matrix size was reduced to 256 × 256 for this experiment. Wretenberg et al. utilized the CT-IMA protocol with rotational stress and varus–valgus stress for knee implants, highlighting protocol optimization for visualization but without detailed dose specifics. Sandberg et al. [27] applied the CT-IMA protocol and reported a relatively high dose of approximately 4.5 mSv per scan, justified by superior sensitivity and specificity outcomes compared to standard radiography.

### 3.4. CT Detection Accuracy for Loosening

A summary of the CT diagnostic accuracy is shown in Table 2.

The first study that examined implant loosening with a provocation CT-based analysis was the one conducted by Berger et al. [24] in 1996. They compared three groups of patients with cementless hip stems: in patients with X-rays positive for loosening, the provocation CT detected 100% of loosening confirmed intraoperatively; in patients with X-rays inconclusive for loosening, the provocation CT detected 20/22 cases of loosening with a sensitivity of 91.6% and a specificity of 90%, with a positive predictive value of 91.6%; patients with recent THA were used as a control group, and provocation CT did not detect any movement. This study underlines the importance of provocation CT in case of equivocal X-rays, potentially avoiding unnecessary revision surgeries.

Reinus et al. [25] included cemented stems in their study and found that provocation CT had a 67% sensitivity, a 100% specificity, and a 75% accuracy in detecting aseptic loosening.

Olivecrona et al. [18], through the CT-IMA protocol, found 100% specificity and good sensitivity, detecting three out of four cups that were loose intraoperatively and not detecting movement in cups that were not loose.

Wretenberg et al. [17]’s approach to knee implants using CT-IMA confirmed CT’s effectiveness in detecting instability and correcting wrong X-rays diagnoses. In fact, of the 22 TKAs with radiolucent lines in X-rays, CT-IMA detected loosening in only 8 implants: 14 revision surgeries were avoided. Sandberg et al. [27] reinforced these findings by demonstrating significantly enhanced sensitivity compared to standard radiographs: CT-IMA detected loosening in 7/9 cups (X-ray in 1/9), 8/10 stems (X-ray in 1/10), and 1 TKA (X-ray also detected 1). In 2022, the same authors showed that standard X-ray was incorrect for 11 cups and 4 stems, while CT-IMA was incorrect for only 4 cups and 0 stems.

None of the clinical studies compared CT protocols with RSA. Eriksson et al. [31], in 2019, conducted a study on a 3D Hip Phantom utilizing 14 different CT protocols. All protocols produced scans in which the numerical data were sufficient for a migration analysis at least as precise as would be expected using RSA. A protocol with an effective dose of 0.70 mSv was then shown to be applicable in a pilot patient.

## 4. Discussion

This systematic review evaluated the current evidence on the use of provocation CT-based analysis in THA and TKA for the diagnosis of aseptic loosening of implants. Across six studies involving a total of 198 patients, the findings consistently support the utility of dynamic CT imaging as a reliable, sensitive, and specific diagnostic modality, particularly when traditional radiographic methods yield inconclusive results.

Dynamic CT-based analysis has increasingly gained attention for diagnosing aseptic loosening following hip and knee arthroplasty, potentially offering an advantageous combination of precision and feasibility compared to traditional imaging methods. RSA, the traditional gold standard, can provide precise detection of early implant migration, yet its clinical implementation is hindered by the need for tantalum markers, specialized equipment, and higher procedural costs [15]. Broden et al. [15] had already shown in 2020 that CT scans could detect tantalum bead and implant movement with a precision of 0.07–0.31 mm in translation and 0.20–0.39° in rotation, representing a good alternative to RSA with a low dose ranging from 0.2 to 2.3 mSV, but they did not use any provocation modality. In 2021, the same authors [32] demonstrated that CT could be equivalent to RSA in detecting translation of cemented cup loosening, with a precision in the range of 0.10–0.16 mm compared to 0.09–0.26 mm, and better in detecting rotation, with a precision of 0.21–0.31° compared to 0.43–1.69° for RSA, but again they did not use any provocation method for their studies. These studies demonstrate that CT scanning can perform better than RSA in implant loosening detection, but it is not cost-effective since the use of tantalum beads must still be considered.

Therefore, provocation CT-based methods, leveraging mechanical stress to assess implant stability dynamically, have been developed as a practical, marker-free alternative to RSA with promising results [33].

Since RSA is not feasible and cost-effective in clinical routine practice, diagnosing loosening of a prosthesis with routine plain X-ray is the standard method used today, and it usually depends on the disclosure of linear osteolysis, which is the most common cause of aseptic loosening [6]. Osteolysis is hard to diagnose using only planar radiographs, and it can cause late diagnosis [7,8,9].

Across the studies reviewed in this systematic review, detection accuracy for implant loosening varied yet consistently underscored CT analysis’s superior sensitivity and specificity over conventional plain X-ray [26]. The heterogeneity of the studies does not allow it to be stated in which cases the use of provocation CT in clinical practice is justified, but in every scenario it showed superiority to standard X-ray.

Collectively, we can affirm that these studies validate provocation CT-based analysis as a sensitive and specific diagnostic modality capable of early detection of implant loosening, thus potentially improving clinical decision-making and patient outcomes, avoiding unnecessary revision surgeries.

The variability in reported radiation doses between the studies included in the review (ranging from 2.5 mSv to 4.5 mSv per scan) emphasizes the need for protocol standardization. Optimizing scanning parameters to balance diagnostic quality with minimized radiation exposure remains a critical issue for clinical adoption [34]. Future research should establish universally accepted low-dose CT protocols, potentially incorporating advanced techniques such as metal artifact reduction algorithms and adaptive dose modulation to ensure patient safety without compromising diagnostic performance.

Cost considerations were directly addressed only by Berger et al. [24], who highlighted the relative simplicity and cost-effectiveness of CT techniques compared to alternative diagnostic methods like bone scintigraphy or invasive arthrograms. The rapid and minimally invasive nature of provocation CT potentially reduces overall healthcare costs by minimizing unnecessary revision surgeries resulting from inconclusive conventional imaging.

In 2023, Lovera et al. [35], through a health-economic model, investigated the cost-effectiveness of using provocation CT-IMA to follow up suspected aseptic loosening when a diagnosis with an initial X-ray was not conclusive, compared with a diagnostic pathway with only X-ray follow-up. They demonstrated that the diagnostic pathway using CT-IMA after an inconclusive X-ray for suspected aseptic loosening was cost-effective compared with a pathway with X-ray follow-up (SEK 99,681, compared with the SEK 500,000 threshold). However, broader and more comprehensive economic evaluations are needed to establish robust evidence regarding cost-effectiveness across diverse healthcare systems.

A major strength is that this systematic review is the first to comprehensively assess CT-Based Implant Motion Analysis specifically for diagnosing aseptic loosening in hip and knee arthroplasty, filling a significant gap in the existing orthopedic literature. This systematic review rigorously adhered to the PRISMA guidelines, thoroughly evaluating the literature over an extensive temporal range. The comparative analytical approach across the various study protocols provided in-depth insights into the diagnostic precision, potential practical applicability, and clinical relevance of dynamic CT. The review’s inclusion of both hip and knee arthroplasty studies enhances its broader applicability, offering valuable guidance for orthopedic clinical decision-making. Despite these strengths, several limitations exist. The limited overall sample size (n = 198 patients) and heterogeneity in the study designs and protocols constrain the generalizability. Inconsistent reporting of technical parameters and radiation dosimetry further complicates a standardized interpretation. Most of the studies were single-center studies with small samples and potential variability in operator expertise, equipment, and software, affecting diagnostic consistency. Two of the studies included were performed in 1996 and so with very different technology. The absence of long-term outcome data limits the evaluation of prognostic utility. Moreover, no study directly compared provocation CT-based analysis and the current gold standard, RSA, and none of the studies was a level I study.

The current evidence is still too weak to justify the routine use of this method.

Future research directions include conducting multicenter, prospective studies with standardized protocols and detailed dosimetric reporting. Exploring the integration of artificial intelligence-driven image analysis techniques could further enhance diagnostic precision and consistency. Comprehensive economic evaluations are necessary to confirm cost-effectiveness, ultimately guiding the establishment of standardized international clinical guidelines for the routine implementation of provocation CT methods in orthopedic practice.

## Figures and Tables

**Table 1:** Study characteristics. CT-Implant Movement Analysis (CT-IMA) is a specific provocation CT protocol (see Section 3.3).

Author, Year	Study Type	Level of Evidence	Country	No. of Patients	Age	Sex (F)	Studied Joint	Studied Component	Protocol	Main Findings
Berger et al., 1996 [24]	Case–control	3	USA	50	Unknown	Unknown	Hip	Cementless stems	Provocation CT	CT effective in detecting loosening, especially when X-ray is equivocal
Reinus et al., 1996 [25]	Cohort	4	USA	16	Mean: 65 (range unknown)	Unknown	Hip	3 cemented stems, 13 cementless stems	Provocation CT	CT effective in detecting loosening
Olivecrona et al., 2008 [18]	Cohort	3	Sweden	10	Mean: 52 (53–79)	4	Hip	Cups	CT-IMA	CT effective
Wretenberg et al., 2021 [17]	Cohort	3	Sweden	40	Mean: 68 (54–86)	23	Knee	37 primary TKAs, 3 revision TKAs	CT-IMA	CT more effective than X-ray
Sandberg et al., 2021 [27]	Retrospective cohort	4	Sweden	10	Mean: 57 (range unknown)	4	Hip and knee	9 cups, 9 stems, 1 knee	CT-IMA	CT more effective than X-ray
Sandberg et al., 2022 [26]	Retrospective cohort	4	Sweden	72	Mean: 69 (40–85)	49	Hip	Cups and stems	CT-IMA	CT more effective than X-ray

**Table 2:** CT protocols and detection accuracy.

Author, Year	Stress Modality	CT Protocol Specifics	Radiation Doses	CT Detection Accuracy
Berger et al., 1996 [24]	Maximum internal and external rotation through a leg holder	2 axial CTs of the stem and the femoral condyles to see differences in the stem version angle between the 2 CTs	Unknown	When X-ray was in doubt regarding loosening, provocation CT showed 91.6% sensitivity, 90% specificity, and a PPV of 91.6%
Reinus et al., 1996 [25]	Maximum internal and external rotation	2 axial CTs of the stem and the femoral condyles to see differences in the stem version angle between the 2 CTs	Unknown	CT showed 67% sensitivity, 95% c.i.: 35–90; 100% specificity, 95% c.i.: 40–100; 75% accuracy, 95% c.i.: 48–93
Olivecrona et al., 2008 [18]	Maximum internal and external rotation maintained through sandbags	Semi-automated software overlays the bone from both CTs. This enables the viewer to achieve an enhanced view of any displacement-induced movement of the implant relative to the bone in which it should be anchored	2.5–3.5 mSv per scan	Of the cups that were loosened at surgery, 3 out of 4 were detected by CT
Wretenberg et al., 2021 [17]	Maximum internal and external rotation maintained through sandbags	Semi-automated software overlays the bone from both CTs. This enables the viewer to achieve an enhanced view of any displacement-induced movement of the implant relative to the bone in which it should be anchored	3.5 mSv per scan	Of the 22 knees that showed radiolucent lines on X-ray, CT showed loosening in only 8. In the 18 knees with negative X-rays, CT was negative too
Sandberg et al., 2021 [27]	Maximum internal and external rotation maintained through sandbags	Semi-automated software overlays the bone from both CTs. This enables the viewer to achieve an enhanced view of any displacement-induced movement of the implant relative to the bone in which it should be anchored	Unknown	CT showed loosening on 7/9 cups, 6/10 stems, and 1/1 knee, while X-ray showed loosening in 1/9 cups, 1/10 stems, and 1/1 knee
Sandberg et al., 2022 [26]	Maximum internal and external rotation maintained through sandbags	Semi-automated software overlays the bone from both CTs. This enables the viewer to achieve an enhanced view of any displacement-induced movement of the implant relative to the bone in which it should be anchored	4.5 mSv per scan	X-ray was incorrect for 11 cups and 4 stems (sensitivity: 59%, 95% c.i.: 35–82; specificity: 85%, 95% c.i.: 74–96), CT was incorrect for 4 cups and 0 stems (sensitivity: 77%, 95% c.i.: 56–97; specificity: 100%, 95% c.i.: 100–100)

## Abbreviations

The following abbreviations are used in this manuscript:

THA	Total Hip Arthroplasty
TKA	Total Knee Arthroplasty
RSA	Radiostereometric Analysis
CT	Computer Tomography
CT-IMA	Computer Tomography-Implant Movement Analysis
PRISMA	Preferred Reporting Items for Systematic Reviews and Meta-Analyses
PICO	Population, Intervention, Comparison, Outcome
ROBINS-I	Risk of Bias in Non-randomized Studies of Interventions
RCT	Randomized Controlled Trial
mSv	Millisievert (unit of radiation dose)
SEK	Swedish crowns
